# Safety Profile of SARS-CoV-2 Vaccination in Patients with Lupus Nephritis: A Retrospective Study

**DOI:** 10.3390/jcm14020406

**Published:** 2025-01-10

**Authors:** Dimitra Petrou, Smaragdi Marinaki, Pelagia Kriki, Sofia Flouda, Aliki Venetsanopoulou, Paraskevi Voulgari, Aggeliki Sardeli, Konstantinos Drouzas, Stylianos Panagoutsos, George Liapis, Harikleia Gakiopoulou, Sophia Lionaki

**Affiliations:** 1 Division of Nephrology, 2nd Department of Internal Medicine, Attikon University Hospital, School of Health Sciences, National and Kapodistrian University of Athens, 12462 Athens, Greece; 2 Department of Nephrology and Renal Transplantation, Laiko Hospital Athens, School of Health Sciences, National and Kapodistrian University of Athens, 11527 Athens, Greece; 3 Department of Nephrology, University Hospital of Alexandroupolis, School of Health Sciences, Democritus University of Thrace, 68100 Alexandroupolis, Greece; 4 Department of Rheumatology, 4th Department of Internal Medicine, Attikon University Hospital, School of Health Sciences, National and Kapodistrian University of Athens, 12462 Athens, Greece; 5 Department of Rheumatology, University Hospital of Ioannina, School of Health Sciences, University of Ioannina, 45500 Ioannina, Greece; 6 Department of Pathology, School of Health Sciences, National and Kapodistrian University of Athens, 11527 Athens, Greece

**Keywords:** lupus nephritis, SARS-CoV-2 vaccination, adverse events, relapse

## Abstract

**Objectives:** Vaccination against SARS-CoV-2 has been vital in alleviating the spread of the recent pandemic. We aimed to estimate the frequency and type of adverse events related to SARS-CoV-2 vaccine in patients with lupus nephritis (LN), and assess its impact, if any, on the risk of subsequent reactivation of nephritis. **Methods:** This was a retrospective, multicenter study which included patients with biopsy-proven LN, who had received at least one vaccine dose. Patients who ended up with end-stage kidney disease (ESKD) prior to vaccination or were diagnosed with LN after vaccination were excluded. Adverse events, systemic or local, COVID-19 outcomes (full recovery, death, or long COVID-19), outcome of LN (remission, refractory disease, relapse, ESKD or death), demographics, laboratory measurements, and immunosuppressive regimens were recorded. **Results:** Sixty-seven patients were included. The median age was 33 (20–46) years. Induction therapy for LN was administered to 92.5% of patients and 74.6% received maintenance therapy. Of these, 94.02% were in remission at vaccination. The BNT162b2 mRNA vaccine was administered in 97.01% of cases, with mild systemic adverse symptoms in 28.35% (myalgias 17.91%, headache 13.43%, arthralgias 13.43%, and fever 10.44%) and local adverse effects in 35.82% (pain 25.37%, swelling 13.43%). Overall, among patients in remission upon vaccination, two (3.17%) experienced a LN relapse within 5.75 (±0.25) months, while 75% of those with active disease at vaccination achieved remission within 21 (±2) months. **Conclusions:** SARS-CoV-2 vaccination appears safe for LN patients without serious adverse events occurring, and there is no significant impact in the clinical course of the disease.

## 1. Introduction

Coronavirus disease 2019 (COVID-19) has impacted millions of people worldwide since the World Health Organization declared it a pandemic on 11 March 2020 [1]. Active immunization against SARS-CoV-2 has played a crucial role in reducing infection rates and related morbidity while helping to alleviate the spread of the virus [2,3].

Systemic lupus erythematosus (SLE) is an autoimmune inflammatory rheumatic disease, a chronic autoimmune disorder of uncertain etiology, which is marked by the loss of immune tolerance, leading to the production of autoantibodies and hyperactive B and T cell response [4]. Renal involvement occurs in approximately 50% of patients within the first year of diagnosis and is a major contributor to both morbidity and mortality. Routine monitoring for LN, including the quantification of proteinuria and microscopic urinalysis, is crucial for prompt disease identification and management considering that the clinical presentation of LN can range from asymptomatic hematuria and/or proteinuria to nephrotic syndrome and rapidly progressive glomerulonephritis with impaired renal function [5,6]. Kidney biopsy remains the gold standard for characterizing the type and extent of glomerular injury.

Morbidity and mortality from vaccine-preventable diseases are reported higher in patients with autoimmune diseases, such as SLE, compared to the general population [7,8]. Despite strong recommendations from medical societies, vaccination rates in these patients remain low [9,10]. Patients with LN in particular are at higher risk of severe COVID-19 due to comorbidities like hypertension, impaired kidney function, and immunosuppressive therapy [11,12]. These factors, along with a hypercoagulable state widely described in SARS-CoV-2 infections, increase the likelihood of thrombotic events and worsen outcomes [13]. In order to minimize vaccine hesitancy in this vulnerable population with serious comorbidities, it is crucial to address the concerns and offer clear, evidence-based information [14].

This study aimed to assess the frequency of adverse events following SARS-CoV-2 vaccination in patients with a history of LN, evaluating said adverse events, the potential impacts on kidney function, and the risk of disease recurrence afterwards.

## 2. Materials and Methods

### 2.1. Study Design and Inclusion Criteria

Patients with biopsy-proven LN, who received at least one dose of the SARS-CoV-2 vaccine and had been diagnosed with LN prior to vaccination, were studied retrospectively. Patients who ended up with end-stage kidney disease (ESKD) prior to vaccination or received the diagnosis of LN after vaccination were excluded. We recorded demographics (gender, age, race), histopathological diagnosis of LN by WHO [6], clinical presentation on diagnosis, past medical history beyond LN, immunosuppressive regimens both as induction and maintenance therapy, the first outcome of LN, the type and date of vaccination, number of vaccine doses, the time interval from biopsy to first vaccine dose, the type of immunosuppression on vaccination, the systemic and local adverse events associated with the vaccine, the frequency of LN relapses post vaccination, the glomerular disease activity status, and the laboratory findings at vaccination and at the end of follow-up time. Adverse events were considered as systemic or local. Systemic events included headaches, myalgias, arthralgias, fever, chills, fatigue, diarrhea, nausea, and lymphadenopathy. Local adverse events included pain, swelling, itch, tenderness, allergic reaction, and rash. Episodes of acute kidney injury leading to acute dialysis or ESKD after vaccination were also recorded. The study was conducted using collected data from 3 nephrology and 2 rheumatology centers across Greece.

### 2.2. Characteristics Related to the LN

In addition to the histopathological class of LN, we recorded the type of immunosuppressive therapy, which had been given for LN, i.e., non-specific therapy or immunosuppressive regimens (cyclophosphamide, glucocorticoids, mycophenolate mofetil, calcineurin inhibitor, rituximab, anti-CD-40 ligand, and azathioprine). Immunosuppressive therapy was recorded from the time of LN diagnosis to the end of follow-up. Furthermore, information regarding the 1st outcome of LN following diagnostic biopsy and the appropriate treatment was collected in all patients, which might be remission, or refractory disease (dialysis patients were excluded from this cohort). The activity status of LN at vaccination and afterwards i.e., at the end of follow-up, was also recorded. Patients who experienced an alteration in the activity status from remission to active disease were considered to have a relapse of LN. In this regard, the time to relapse was estimated from the first dose of vaccination to the documentation of LN relapse. In addition, the outcome of patients who were active at vaccination was recorded i.e., remission, ESKD or death, until the end of follow-up. Acute dialysis was defined as the need for dialysis in the context of acute kidney injury. Data collection prompted research coordinators to ask participants and to review medical records in each institution in order to collect all information regarding adverse events, hospitalizations, and the course of LN.

Definitions of the outcomes of LN, remission, refractory disease, active disease, and relapse were noted according to the Kidney Diseases Improving Global Outcomes [6]. The complete remission of LN was defined as both a reduction in proteinuria <0.5 g/g, measured as the protein-creatinine-ratio (PCR) from a 24 h urine collection, and the stabilization or improvement in kidney function [6]. Partial remission of LN was defined as both a reduction in proteinuria by at least 50% and to <3 g/g, measured as the PCR from a 24 h urine collection, and the stabilization or improvement in kidney function [6]. Refractory disease was defined as a failure to achieve a partial or complete response within 6–12 months of therapy initiation [6]. A relapse of LN was defined as a progressive decline of renal function with proteinuria reduction less than 50% and >3g/g [6]. Activity status alteration might include a change from active disease to remission or a change from remission status to active disease i.e., relapse. The observation period started at vaccination (date of administration of 1st dose) and ended at the time of the most recent visit to the glomerular clinic post vaccination. End-stage kidney disease was defined as the chronic requirement of dialysis. Acute dialysis was defined as the need for dialysis for less than three months. The estimated glomerular filtration rate (eGFR) was calculated using the Chronic Kidney Disease Epidemiology Collaboration (CKD-EPI) equation [15]. Laboratory measurements before and after vaccination, including laboratory findings obtained during the closest visit prior to vaccination, the first visit after vaccination, and at the end of follow-up, were collected.

Descriptive statistics include *n*, percent, proportions, mean, s.d, and median (range) regarding demographics and clinical characteristics of the participants. An unpaired T-test was used to compare values of laboratory measurements before and after vaccination as well as other parameters. Analyses were conducted using SAS 9.1 (SAS Institute, Cary, NC, USA). Exact *p*-values are reported with a two-sided *p*-value of 0.05 or less considered statistically significant.

## 3. Results

### 3.1. Description of Study Cohort and Baseline Characteristics

A total of 67 patients with a known history of biopsy-proven LN were included in the study. Overall, the median age of the patients was 33 (20–46) years and 10 (14.92%) were males. They were all of Greek origin and a White/Caucasian race. A small portion of the studied population (7/67 cases) were classified as class I or II (0.14%), while 13/67 cases fell under class III (19.4%). The largest group in this cohort was class IV which contained 22 cases (32.8%). This indicates that class IV was the most prevalent in this cohort. There were 10 individuals in class V (14.9%). There were also 11 cases with overlap between classes IV and V (16.4%) and 2 cases with overlap between classes III and V. Finally, there was one case (1.5%) with lupus podocytopathy and one (1.5%) with thrombotic microangiopathy (Table 1).

Regarding the immunosuppressive regimens used for induction and maintenance therapy in our cohort, 62 (92.53%) patients received induction therapy, including 58 patients (86.56%) who received glucocorticoids, 41 (61.19%) treated with cyclophosphamide, 14 (20.89%) treated with mycophenolate mofetil, 6 (8.95%) treated with rituximab, and 3 (4.47%) with a calcineurin inhibitor (Table 1). In terms of the maintenance phase, the vast majority, 50 patients (74.62%), were treated with mycophenolate mofetil, while 17 patients (25.37%) were maintained on glucocorticoids dual therapy with either mycophenolate mofetil or azathioprine. In total 10 patients (14.92%) received azathioprine, 3 patients (4.47%) calcineurin inhibitors and 2 patients (2.98%) continued cyclophosphamide during the maintenance treatment. Only 2 patients (2.98%) were maintained on rituximab, while a total of 8 patients (11.94%) received rituximab during the course of the disease as either induction or maintenance therapy. Most patients, 65 (97.015%), had achieved remission as their first outcome of LN. Among them, 60 (89.55%) achieved complete remission and 5 (7.46%) partial remission. Only 2 patients (2.98%) had refractory disease (Table 1).

### 3.2. LN Activity and Immunosuppressive Status upon Vaccination

The majority of patients in this cohort (97.01%) received the BNT162b2 vaccine. Two patients received the ChAdOx1 nCoV-19 vaccine (2.98%). The average number of vaccine doses was 2.5 (±0.5). The average time from kidney biopsy to the first vaccine dose was 90.05 (±12.58) months. At vaccination, 51 individuals (76.11%) were on an immunosuppressive treatment of any type (Table 2).

### 3.3. Adverse Events Associated with Vaccination

Both systemic and local adverse events associated with SARS-CoV-2 vaccination were recorded and 19 (28.35%) patients experienced at least one systemic side effect. Myalgias were the most common systemic adverse event, affecting nearly 12 (18%) participants. Headache and arthralgias were also relatively common, affecting nine (13.43%) participants. Low grade fever occurred in seven (10.44%) patients (7/67), while chills and fatigue were both reported by a smaller proportion of patients (7.46%). Only one patient (1.49%) reported nausea and two (2.98%) lymphadenopathy. Local adverse events were more frequent, accounting for 24 participants (35.82%) with local pain being the most common i.e., documented in 17 (25.37%) participants. Swelling was reported by nine (13.43%) individuals. No tenderness, itch, allergic reaction, or rash were documented. No renal adverse events or acute kidney injury were noted as a result of vaccination (Table 2).

A further analysis of subgroups according to different treatment regimens was conducted to reveal their impact on the systemic and local side effects post-vaccination (Table 3). Regarding induction therapy, 20.68% of patients on glucocorticoids experienced systemic side effects post-vaccination, while 34.48% experienced local side effects. In the cyclophosphamide group, 29.26% of individuals experienced systemic side effects and 31.7% experienced local side effects. Mycophenolate mofetil showed an incidence of 28.57% for systemic and 35.71% for local adverse events post-vaccination, while 16.66% of patients on rituximab experienced systemic side effects and 33.33% experienced local side effects. The calcineurin inhibitor group demonstrated no systemic side effects but 33.33% experienced local side effects when applied as an induction treatment. Regarding maintenance therapy, cyclophosphamide showed balanced systemic (50%) and local (50%) side effects post-vaccination. Only 5.88% of patients on corticosteroids experienced systemic side effects, while 29.41% experienced local adverse events. Individuals on mycophenolate mofetil documented systemic side effects in 28% of cases, while local ones were reported in 36% of cases. Calcineurin inhibitors, azathioprine, and rituximab when applied as maintenance therapy demonstrated equal rates of systemic and local side effects (33.33%, 40%, and 50%, respectively). On the other hand, the use of rituximab ever, either as an induction or maintenance therapy, demonstrated systemic advese events in 25% of patients and local ones in 37.5% (Table 3). An additional analysis of subgroups based on the presence or absence of immunosuppression upon vaccination was performed to assess its impact on vaccine-related side effects. Of those on immunosuppressive therapy (51/67, 76.11%), 29.41% experienced systemic side effects, while 25% of individuals in the off-immunosuppression group reported similar symptoms. Local reactions were observed in 29.41% of cases in the immunosuppressed group, compared to 43.75% in the non-immunosuppressed group (Table 3).

The assessment of LN activity status at the time of 1st vaccine dose is shown in Table 4. Notably, 63 (94.02%) patients were in remission, while 4 (5.97%) had active disease. In the on-immunosuppression group, a small proportion of patients had active disease (7.84%), whereas no patients off immunosuppression had active disease at vaccination (Table 5). Among those in remission upon vaccination, two (3.17%) patients experienced a relapse of LN afterwards, within a median time of 5.75 (±0.25) months. Both were females, had membranous LN and lupus podocytopathy as histopathological diagnoses, and were on immunosuppression upon vaccination. The average time from kidney biopsy to the first vaccine dose was 40 (±32) months. The follow-up time for the total population was 21 (±2) months from vaccination. Regarding LN activity status at the end, 65 (97.01%) patients were in remission and 2 (2.985%) remained with active LN disease. Among patients who were active at vaccination, three (75%) achieved remission by the end of the observation period, with all of them being on immunosuppressive therapy (Table 5).

### 3.4. Laboratory Measurements Before and After Vaccination

Laboratory data were recorded including hematologic (hemoglobin, white blood cell count, neutrophil count, lymphocyte count, and neutrophil-to-lymphocyte ratio), inflammatory indexes (serum lactate dehydrogenase—LDH and C-reactive protein—CRP), and renal function markers (serum creatinine, eGFR, 24 h urinary protein excretion, and max urine RBC per high power field) (Table 6 and Table 7).

After implementing an unpaired T-test, most of the parameters demonstrated significant changes between the “Before vaccination” and “After vaccination” time points. Hemoglobin decreased from 11.95 to 11.15 (*p*-value < 0.0001), white blood cell count decreased from 5865 to 4605 (*p*-value < 0.0001), neutrophil count decreased from 4102 to 3139 (*p*-value < 0.0001), lymphocyte count decreased from 1364.5 to 1144.5 (*p*-value < 0.0001), the neutrophil-to-lymphocyte ratio decreased from 3.007 to 2.743 (*p*-value < 0.0001), and LDH decreased from 191 to 183.5 (*p*-value 0.0098). CRP showed no change (*p* value 0.9258). Serum creatinine (mg/dL) increased slightly from 0.64 to 0.725 (*p*-value < 0.0001) but eGFR showed no statistically significant alteration (115 to 115.5 (*p*-value 0.2965)). The maximum number of urine RBCs per high power field remained stable (6 RBC/hpf) and the 24 h urinary protein excretion decreased from 1265 to 657.5 (*p*-value < 0.0001). These results remained consistent after performing comparisons on non-relapsers post-vaccination (Table 7). Even in relapsers, the comparison of parameters before and after vaccination showed no statistically significant changes.

## 4. Discussion

To our knowledge, this is the first reported study specifically focusing on patients with biopsy-proven LN to assess the safety profile of the SARS-CoV-2 vaccination, despite several studies having investigated the effects of vaccination in lupus patients in general [16,17,18,19,20,21,22]. This cohort of patients with LN may be considered a representative one, as a small proportion (14.9%) of cases were males suggesting a gender imbalance typical of LN [6]. A great proportion of patients had diffuse proliferative glomerulonephritis (49.2%), either alone or with membranous LN, suggesting a complex disease with a more severe clinical presentation, requiring intensive therapy, and related to worse renal prognosis. Overall, the high remission rate of 97% suggests that the given treatment regimens were largely effective. We observed a low incidence of both systemic and local adverse events related to the SARS-CoV-2 vaccination in this population. Specifically, we recorded the frequency of systemic adverse events being in 28.35% of patients, most often musculoskeletal symptoms, like myalgias and arthralgias, or a mild headache. These are commonly reported with many medications or vaccines and may suggest a mild immune-mediated reaction [23]. Fatigue, fever, and chills were found less frequently, indicating a mild flu-like response in some individuals. All these effects were typically transient and were resolved on their own. Among local effects, pain at the site of administration was the most common (25.4%), but the overall incidence was low. Importantly, there were no significant local reactions like rash, or itching, which is promising in terms of tolerability [24,25]. Kikuchi et al. studied 90 vaccinated SLE patients, of whom 52.2% had a history of LN. In total, 88.9% of patients reported experiencing adverse reactions within three days following the second vaccine dose, with 73.6% being local effects and 79.2% systemic reactions [16]. The difference in the increased rate of vaccine-related adverse events in that study might be explained by the fact that the patient recruitment in the Kikuchi et al. study took place between June and October 2021, a period shortly after the approval of the BNT162b2 vaccine, which led to an increased global focus on monitoring and vigilance [2,16]. The international VACOLUP study was a multi-ethnic study across 30 countries involving 696 SLE patients, 240 (34.5%) of whom had kidney involvement [17]. The primary outcome was the occurrence of side effects, including flare. Adverse events following SARS-CoV-2 vaccination were reported in 50% of the participants. Local pain was reported in 45%, while headache was present in 35% of participants. Wang et al. also aimed to assess the seroreactivity to SARS-CoV-2 vaccination and the associated adverse events in patients with autoimmune diseases compared to healthy individuals [18]. The study included 60 SLE patients, of whom 18 had LN. Among outcomes of interest, systemic adverse events following SARS-CoV-2 vaccination were reported in 25% of participants, while myalgias (7%), fever (5%) and fatigue (3.3%) were reported at almost similar rates to those observed in our study.

Immunosuppressive medications influence vaccine-related side effects in distinct ways, with systemic side effects being more effectively suppressed compared to local reactions in many treatment regimens. Glucocorticoids and rituximab, used in induction therapy in our study, were associated with reduced systemic side effects, indicating that these therapies dampen the overall immune response, resulting in fewer generalized adverse reactions. Rituximab and cyclophosphamide demonstrated lower rates of systemic reactions while maintaining moderate local responses. In contrast, mycophenolate mofetil and azathioprine exhibited a more balanced suppression of both systemic and local side effects. In addition, patients receiving immunosuppressive therapy at the time of vaccination experienced similar rates of systemic side effects compared to those not on immunosuppression. However, individuals who were off immunosuppression at the time of vaccination exhibited a higher incidence of local side effects, potentially due to a more robust local immune response. These findings were in accordance with other published studies [17,18,19,20,21].

Regarding the probability of disease re-activation among remitted patients, we found that only two individuals (3.2%) experienced a relapse of LN after vaccination, within 5.75 months. Previous reports have shown that flares after vaccine administration range from 3% to 20% in patients with SLE [16]. Kikuchi et al. found that the SLE Disease Activity Index (SLEDAI) increased modestly but significantly after vaccination, with 14.4% of patients experiencing flares, including 4.4% with severe flares (three with nephritis) [16]. The flare rate was higher in vaccinated patients compared to the unvaccinated controls. Multivariate analysis identified a high SLEDAI and the presence of anti-dsDNA antibodies as factors associated with flares, suggesting that residual disease activity and serological activity prior to vaccination increase the risk of disease flare. Izmirly et al. assessed immune response and disease status in SLE patients following SARS-CoV-2 vaccination [19]. Their study included 90 SLE patients, 40 (44%) of whom had LN. Nine of the seventy-nine patients (11.4%) experienced a post-vaccination flare, with all but one classified as mild to moderate. One severe flare involved a renal manifestation, characterized by recurrent proteinuria after the second dose of BNT162b2 vaccine. Mok et al. studied post-vaccination flares in 914 SLE patients, 52% with a history of LN [22]. Among 449 vaccinated patients (227 with LN), 8.2% experienced flares within a period of 6 weeks follow-up time (34 mild/ moderate, 3 severe), of whom 4% were renal manifestation. However, in matched unvaccinated controls, 6.2% had flares (17 mild/moderate, 11 severe). Logistic regression showed that active lupus serology (OR 2.63), a history of arthritis (OR 2.71), and discoid lessions (OR 4.73) were associated with flares post-vaccination. Vaccination was not significantly linked to increased flares, but patients with active serology or a history of arthritis/discoid lesions were more likely to flare [22]. The international VACOLUP study by Felten et al. with 696 SLE patients also recorded a 3% post-vaccination flare rate, of whom only 10% had a renal involvement [17]. The low relapse rate in our study suggests that vaccination may not significantly increase the risk of disease flare in this specific population, though short-term monitoring would be wise, especially in the first few months after vaccination.

The BNT162b2 vaccine was administered in 97.01% of cases in our study. The absence of mRNA-1273 and Janssen vaccines in this cohort may reflect a limited preference at the time of vaccination. The predominant choice of the BNT162b2 vaccine was due to its greater availability and clinical preference for this population; these findings are consistent with other studies [16,17,19,20]. On average, individuals received 2.5 vaccine doses. This aligns with typical vaccination strategies, which started with two doses, while additional boosters became standard over time [2,6]. The average time from biopsy to the first vaccine dose was roughly 7.5 years, suggesting that this cohort consisted of people who have had long-term management of their disease, likely meaning they have been stable for several years. Yet, a long period from biopsy to vaccination might indicate that individuals with controlled kidney disease is a result of the fact that the pandemic occurred years after the diagnostic biopsy. Therefore, the majority of individuals were in remission at vaccination, which could lead to a more favorable vaccine response compared to those with active disease.

Notably, a few patients had active disease at vaccination, of whom 75% achieved remission by the end of the follow-up period. This is a strong argument that vaccination is beneficial even for individuals with active disease, leading to an improvement in renal disease. The follow-up time of 21 months was long enough to gauge the initial effects of the vaccination and to monitor disease status over a reasonable period. It is important to highlight that the follow-up periods in other studies were relatively shorter than 21 months (Mok et al.: 6 weeks, Kikuchi et al.: 2 months, Izimirly et al.: 2 months, and Felten et al.: 19 months) [16,17,19,22]. Given that most patients remained in remission over this period in our study, the long-term outcomes of vaccination appear favorable. At the end of follow-up, most patients in both groups were in remission and even the off-immunosuppression group maintained a perfect remission rate (100%). This suggests that immunosuppression may help maintain remission in most patients, but a small subset may still experience flares. On the other hand, the off-immunosuppression group had a higher likelihood of sustained remission, but with fewer patients to evaluate, this finding may not be as generalizable.

Hematologic markers showed a modest reduction in white blood cell subtypes, with a slight drop in haemoglobin, indicating a mild alteration in the immune and hematologic systems post-vaccination in this population. Inflammatory markers showed no significant change and renal function markers showed minor fluctuations. No significant change in the urine sediment was documented, while protein excretion improved, suggesting no major adverse effects on kidney function.

The limitations of this study include the relatively small sample size and the retrospective design and thus it may have been underpowered to detect rare adverse events. However, the disease is rare itself. The lack of male patients and the exclusive White/Caucasian population due to geographic/ethnic factors within our country limit the study’s applicability to other racial or ethnical groups. Larger, more diverse, multinational and multiracial studies would be needed to further validate these findings. Unfortunately, due to the retrospective nature of this study, data on SLE serological markers, the SLEDAI score, and the seroprevalence of IgG and IgM antibodies against SARS-CoV-2 pre/post-vaccination were not available for inclusion. While side effects in this cohort were infrequent and largely mild, longer-term monitoring might show potential delayed adverse events. The time to relapse was relatively short, indicating that although relapse is uncommon, it may still occur within a few months post-vaccination. Therefore, close monitoring in the months following vaccination is necessary, especially in those with active disease at the time of vaccination. Further studies with extended follow-up durations are more likely to identify delayed adverse events with increased accuracy and certainty. Data on long-term side effects would further solidify the safety profile of the vaccines in these populations.

## 5. Conclusions

In conclusion, according to the findings from this study, vaccination against SARS-CoV-2 was associated with favorable outcomes in patients with LN. No serious adverse events were recorded, while we observed maintenance of high rates of sustained remission after vaccination and the achievement of remission in patients who were active at vaccination. Probably, the immunosuppressed population may experience a lower incidence of systemic reactions, although further investigation is needed to explore the implications for vaccine efficacy in this group. The study’s findings provide reassurance for vaccine safety, though a larger, more diverse sample is needed for more robust conclusions.

## Figures and Tables

**Table 1:** Demographics and baseline characteristics of the entire cohort of patients with LN.

Parameter (N (%) or Mean ± sd)	Study Sample (N = 67)
Age at dx (years)	33 (13)
Male sex	10 (14.92)
Race	White/Caucasian 67 (100)
**Class by WHO**	
I, II	7 (0.14)
III	13 (19.40)
IV	22 (32.83)
V	10 (14.92)
VI	0
IV + V	11 (16.41)
III + V	2 (2.98)
Lupus podocytopathy	1 (1.49)
Thrombotic microangiopathy	1 (1.49)
**Induction therapy**	62 (92.53)
Glucocorticoids	58 (86.56)
Cyclophosphamide	41 (61.19)
Mycophenolate mofetil	14 (20.89)
Rituximab	6 (8.95)
Calcineurin inhibitor	3 (4.47)
**Maintenance therapy**	62 (92.53)
Cyclophosphamide	2 (2.98)
Corticosteroids	17 (25.37)
Mycophenolate mofetil	50 (74.62)
Azathioprine	10 (14.92)
Calcineurin inhibitor	3 (4.47)
Rituximab	2 (2.98)
Rituximab ever	8 (11.94)
**First outcome of LN**	
Remission	65 (97.015)
Complete remission	60 (89.55)
Partial remission	5 (7.46)
Treatment resistant	2 (2.985)

**Table 2:** Vaccine-related parameters and adverse events associated with vaccination.

Parameter (N (%) or Mean ± sd)	Study Sample (N = 67)
**Vaccine type**	
BNT162b2	65 (97.01)
mRNA-1273	0
Janssen	0
ChAdOx1 nCoV-19	2 (2.98)
Number of vaccine doses	2 (0.5)
Time to 1st vaccine dose from biopsy (months)	90.05 (12.58)
Immunosuppression on vaccine	51 (76.11)
**Systemic side effect (any)**	19 (28.35)
Headache	9 (13.43)
Myalgias	12 (17.91)
Arthralgias	9 (13.43)
Fever	7 (10.44)
Chills	5 (7.462)
Fatigue	5 (7.462)
Diarrhea	0
Nausea	1 (1.492)
Lymphadenopathy	2 (2.985)
**Local side effects (any)**	24 (35.82)
Pain	17 (25.37)
Swelling	9 (13.43)

**Table 3:** Impact of different treatment regimens and immunosuppression, upon vaccination, on systemic and local side effects.

Parameter (N (%) or Mean ± sd)	Systemic Side Effects	Local Side Effects
**Type of treatment**		
**Induction therapy** **(N = 62, 92.53)**		
Glucocorticoids (N = 58, 86.56)	15/58 (20.68)	20/58 (34.48)
Cyclophosphamide (N = 41, 61.19)	12/41 (29.26)	13/41 (31.70)
Mycophenolate mofetil (N = 14, 20.89)	4/14 (28.57)	5/14 (35.71)
Rituximab (N = 6, 8.95)	1/6 (16.66)	2/6 (33.33)
Calcineurin inhibitor (N = 3, 4.47)	0/3 (0)	1/3 (33.33)
**Maintenance therapy** **(N = 62, 92.53)**		
Cyclophosphamide (N = 2, 2.98)	1/2 (50)	1/2 (50)
Glucocorticoids (N = 17, 25.37)	1/17 (5.88)	5/17 (29.41)
Mycophenolate mofetil (N = 50, 74.62)	14/50 (28)	18/50 (36)
Azathioprine (N = 14, 14.92)	4/10 (40)	4/10 (40)
Calcineurin inhibitor (N = 3, 4.47)	1/3 (33.33)	1/3 (33.33)
Rituximab (N = 2, 2.98)	1/2 (50)	1/2 (50)
Rituximab ever (N = 8, 11.94)	2/8 (25)	3/8 (37.5)
**Immunosuppression at vaccination**		
On-immunosuppression (N = 51, 76.11))	15/51 (29.41)	15/51 (29.41)
Off-immunosuppression (N = 16, 23.88)	4/16 (25)	7/16 (43.75)

**Table 4:** Lupus nephritis activity status at vaccination and disease related outcomes at the end of follow-up period.

Parameter (N (%) or Mean ± sd)	Study Sample (N = 67)
**LN activity status at vaccination**	
Remission	63 (94.02)
Active	4 (5.97)
**LN Relapse after vaccination among those in remission**	2 (3.17)
Time to relapse from vaccination (months)	5.75 (0.25)
**LN Activity status at follow-up end**	
Remission	65 (97.01)
Active	2 (2.98)
Patients who achieved remission after vaccination (among active patients at vaccination)	3 (75)
Follow-up time (months)	21 (2)

**Table 5:** Activity status of LN at the end of follow-up period for patients who were on or off immunosuppression at vaccination.

Parameter (N (%) or Mean ± sd)	On Immunosuppression N = 51	Off Immunosuppression N = 16
**LN activity status at vaccination**		
Remission	47 (92.15)	16 (100)
Active	4 (7.84)	0
**LN Relapse after vaccination**	2 (3.92)	0
Time to relapse from vaccination (months)	5.75 (0.25)	-
**Activity status at follow-up end**		
Remission	49 (96.07)	16 (100)
Active	2 (3.92)	0
Patients who achieved remission after vaccination (among active patients at vaccination)	3 (75)	0
Follow-up time (months)	21 (1)	15.86 (4.5)

**Table 6:** Comparison of laboratory findings before vaccination and at the end of follow-up period.

Parameter (N (%) or Mean ± sd)	Before Vaccination	End of Follow-Up	*p*-Value
Hemoglobin (g/dL)	11.95 (0.15)	11.15 (0.05)	<0.0001
WBC count (/μL)	5865 (465)	4605 (495)	<0.0001
Neutrophil count (/μL)	4102 (202)	3139 (1361)	<0.0001
Lymphocyte count (/μL)	1364.5 (35.5)	1144.5 (44.5)	<0.0001
Neutrophil-to-lymphocyte ratio	3.007 (0.42)	2.743 (0.34)	<0.0001
Serum lactate dehydrogenase (U/L)	191 (23)	183.5 (4.5)	0.0098
C-reactive protein (mg/L)	1.375 (0.625)	1.385 (0.615)	0.9258
Serum creatinine (mg/dL)	0.64 (0.04)	0.725 (0.035)	<0.0001
eGFR (mL/min/1.73 m^2^)	115 (3)	115.5 (2.5)	0.2965
24-h urinary protein excretion (mg)	1265 (735)	657.5 (67.5)	<0.0001
Max urine RBC per high power field	6 (4)	6 (4)	1

**Table 7:** Comparison of laboratory findings before vaccination and at the end of follow-up for non-relapsers.

Parameter (N (%) or Mean ± sd)	Before Vaccination	End of Follow-Up	*p*-Value
Hemoglobin (g/dL)	11.95 (0.15)	11.15 (0.05)	<0.0001
White blood cells count (/μL)	5100 (465)	4605 (495)	<0.0001
Neutrophil count (/μL)	4102 (202)	3139 (1361)	<0.0001
Lymphocyte count (/μL)	1364.5 (35.5)	1144.5 (44.5)	<0.0001
Neutrophil-to-lymphocyte ratio	3.007 (0.64)	2.74 (0.62)	<0.0001
Serum lactate dehydrogenase (U/L)	191 (23)	183.5 (4.5)	0.0110
C-reactive protein (mg/L)	1.375 (0.625)	1.385 (0.615)	0.9269
Serum creatinine (mg/dL)	0.64 (0.04)	0.725 (0.035)	<0.0001
eGFR (mL/min/1.73 m^2^)	115 (3)	115.5 (2.5)	0.3039
24-h urinary protein excretion (mg)	1265 (735)	657.5 (67.5)	<0.0001
Max urine RBC per high power field	6 (4)	6 (4)	1

## Data Availability

The data presented in this study are available on request from the corresponding authors due to privacy and ethical reasons.
